# Spain’s Hesitation at the Gates of a COVID-19 Vaccine

**DOI:** 10.3390/vaccines9020170

**Published:** 2021-02-18

**Authors:** Hans Eguia, Franco Vinciarelli, Marina Bosque-Prous, Troels Kristensen, Francesc Saigí-Rubió

**Affiliations:** 1 Faculty of Health Sciences, Universitat Oberta de Catalunya (UOC), Barcelona 08018, Spain; heguia@uoc.edu (H.E.); mbosquep@uoc.edu (M.B.-P.); 2 SEMERGEN New Technologies Working Group, 28009 Madrid, Spain; fvincia0@rosario.gov.ar; 3 Emergency Hospital Clemente Álvarez, Rosario (Santa Fe) S2002, Argentina; 4 Danish Centre for Health Economics, University of Southern (DaCHE), 5230 Odense, Denmark; trkristensen@health.sdu.dk

**Keywords:** vaccination hesitancy, COVID-19, perception, socio-cultural factors

## Abstract

(1) Background: This study aims to delineate a pattern on vaccine hesitancy in a sample of the Spanish population, considering age groups and status as healthcare workers. (2) Methods: Participants were recruited using Twitter^®^ as a dissemination tool to reach as many respondents as possible in different parts of the Spanish territory. The participants were recruited in a cross-sectional study, which included answering an online questionnaire. Data were collected from 10 September through 23 November 2020. Respondents answered questions asking whether they intended to be vaccinated and provided the main reason for their answers. To estimate associations between vaccination hesitancy and independent variables, we fit Poisson regression models with robust variance. (3) Results: One thousand and two responses were obtained, of which only 731 were validated. One hundred and sixty-four participants stated that they would not be vaccinated (22.43%), of which 20–24% were non-health workers or unemployed, 17.5% physicians, 31.5% other health workers, and almost 35% nurses. Concerns about lack of effectiveness of the vaccination, lack of safety when vaccinating and possibly dangerous adverse effects were the main causes provided. (4) Conclusions: This study indicates that more interventions are needed to achieve better communication with the population and health professionals. Receptiveness to the message of the importance and security of the COVID-19 vaccination could be an important strategy for improving these results.

## 1. Introduction

Vaccination has played a fundamental role in global public health, leading to increased life expectancy [1] while reducing the risk of potentially fatal diseases such as smallpox, which has been eradicated worldwide thanks to vaccination [2]. Although vaccination is considered the most successful method of limiting or eliminating viral infections and spread [3], many people are not convinced of the role of vaccines in immunity, and this results in a decrease in the number of people vaccinated [4]. This also leads to the reemergence of diseases that were previously eradicated in some countries. For example, diphtheria was eradicated 20 years ago from Peru, yet this year several cases have been reported due to lack of vaccination [5].

The rejection of vaccination has led the World Health Organization (WHO) to be concerned about vaccine hesitancy and named it as one of the 10 main global threats in 2019 [6]. However, this is not a new movement. In 1853, a small segment of the population rejected mandatory vaccination of infants because the parents’ decision was not taken into account [7].

Notably, the people most reluctant to be vaccinated are those from low social classes [8], which is precisely the group most susceptible to acquiring a contagious disease [9]. A similar relationship exists with young women (between 18 to 35 years old), who also show some rejection of vaccination and who, in many cases, decide whether their children will be vaccinated [10].

In the US, 49% of the survey participants were receptive to vaccination [3], 31% were unsure and 20% said they would not be vaccinated against COVID-19 [11]. Twenty percent of Canadian survey respondents said that they will not get vaccinated [12], as well as 14% of Italian respondents [13], 26% of French respondents [12], over 50% of English respondents [14] and 14% of Australian respondents [15]. This is consistent with the growing trend of vaccine hesitancy seen in more than 90% of countries [16], especially in Europe [17,18].

After reviewing these data, a question arises: why does this level of uncertainty exist for the future COVID-19 vaccine? A probable set of factors influence the population (factors not only related to this vaccine but also to other vaccines available), such as fear of its safety [3], fear of probable side effects [19], lack of trust in institutions (government, WHO, laboratories) [20], belief it lacks efficacy, belief in conspiracy theories [21] and influence of misinformation. The fear of vaccine safety can be seen in the willingness of parents to vaccinate their children. Interestingly, parents vaccinate their children after a "trial" period (30 days), not immediately, and this amount increases even if six months pass after the start of vaccination [3]. Misinformation can also discourage people from vaccination, and the presence of a biological laboratory in the same city where the virus started spreading could create an image contributing to the growth of conspiracy theories.

Many of the anti-vaccination supporters use social networks to easily and quickly spread their messages and ideologies. On Twitter, a medium and communication platform [22], influencers (opinion leaders, innovators, and celebrities with huge amounts of followers [22]) have frequently tweeted anti-vaccination comments read by thousands of followers.

Many research studies about COVID-19 vaccine hesitancy have been conducted in Europe, the USA and Asia, but not in Spain. It is crucial to determine the population’s attitude towards a COVID-19 vaccination as to manage the COVID-19 pandemic, the vaccine hesitancy factors of the population must be identified. We need more than 70% of people vaccinated in a community to achieve herd immunity [12,23], which means that the vaccine alone is not enough [21].

To devise appropriate policies and preparations for the COVID-19 vaccination, we must first identify the negative perceptions from the population in order to counter them. The wrong policy could create more rejection than affinity. For example, in many countries the possibility of mandatory vaccination has been approached; however, it seems to be inadequate in individual societies due to the increase in anti-vaccine sentiment [11,12]. Therefore, we aim to identify the attitudes or reasons related to the COVID-19 hesitancy in Spain in order to determine adequate public education programs that can be helpful to some extent [12].

To identify the possible factors related to COVID-19 vaccination hesitancy, we have considered Thomson’s “5A” to describe vaccine adoption: access, affordability, awareness, acceptance and activation [24]. Considering these factors will allow us to establish probabilities of refusal to the vaccine even before it is available; moreover, it will allow the establishment of models that can be used to combat vaccine hesitancy.

## 2. Materials and Methods

A self-report design was used in which participants answered an internet-based survey delivered by Twitter that included demographic questions, profession identification, vaccination intention and attitudes towards the COVID-19 vaccination.

To obtain a more geographically diverse sample of the Spanish population, we decided social networks were an adequate source to disseminate our survey because there were more than three million social media posts from January to mid-March 2020 related to COVID-19 vaccination [25]. Twitter was chosen because it is a social network that allows people who are not part of our network of acquaintances to interact and thus avoid bias since it does not take into account degree of empathy, educational level, age, sexual orientation or income. The use of social networks to disseminate the survey will also inform later communication strategies to the population, when assessing the response to our survey.

Some tests are described to measure vaccination attitudes, such as the Vaccination Attitudes Examination (VAX) Scale [26]. However, a short survey different from those used in the literature was designed, addressed specifically to the Spanish public, with short questions and only one with an open response. This was intended to reduce the response time for the participants since longer response times reduces the altruistic participation of the population. Our questionnaire was not incentivized and was short in order to obtain as many answers as possible (see questionnaire in Appendix A). The variables of sex and age were considered necessary to assess whether the type of responses changed according to age or whether there was a willingness of either sex to vaccinate. Profession was also considered to validate the responses of physicians and nurses as health personnel, so they could be compared with the rest of the participants. Finally, the degree of willingness to be vaccinated was asked, offering seven reasons why the respondent does not want to be vaccinated. The last option was open-ended so the participants could provide their own responses (which in many cases was one of the previous choices).

Age groups were established between 18 and 35 years (Group 1), between 36 and 55 years (Group 2), between 56 and 75 years (Group 3), finally those older than 75 years (Group 4).

Data were collected from 10 September 2020 until 23 November 2020. There were 1002 answers collected from the survey; two hundred and fourteen of them were excluded because they were answered from a country other than Spain (for example Peru, Argentina, Mexico, Chile, and the US, among others). Nine responses were excluded because they were completed by participants under the age of 18.

Firstly, we described the sample to establish the main characteristics of the participants. Then, we estimated the prevalence of participants who reported that they would not be vaccinated for the whole sample and for each independent variable. Finally, in order to determine whether there was an association between the intention to be vaccinated and each of the independent variables, we estimated Poisson regression models with robust variance, with their corresponding 95% confidence intervals (95%CI) [27]. All statistical analyses were conducted with STATA 16.

## 3. Results

### 3.1. Sample Profile

The total number of participants from Spain was 731 (44.04% were men; 55.40% were women and 0.54% did not identify their gender). The average age was 50.58 years (between the ages of 21 and 85). Thirty-seven of those surveyed were 49 years old (higher number of participants). The groups with the most respondents were Group 2 with 335 participants (45.82%) and Group 3 with 293 participants (40.08%). In other words, the largest number of participants was between 36 and 75 years old.

### 3.2. Intention to Get Vaccinated

Five hundred and sixty-seven participants or 77.56% of those surveyed were in favour of being vaccinated. Although there were no statistically significant differences, the prevalence of vaccination acceptance was slightly higher in older age groups (Table 1).

#### Reasons for Not Getting Vaccinated

**Figure 1:**
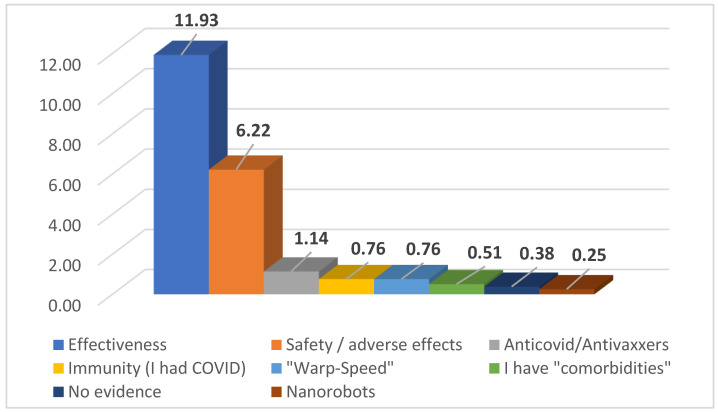
Main reasons for not wanting to get vaccinated (global) in percentage.

One hundred and sixty-four participants or 22.43% of those surveyed stated that they would not be vaccinated. The reasons provided for not being vaccinated were diverse, among them: a lack of effectiveness of the vaccination, a lack of safety when vaccinating or possibly dangerous adverse effects; beliefs that vaccines in general are harmful and/or that COVID-19 does not exist; already had COVID-19 and belief in having immunity; beliefs that these vaccines are not safe because of the speed at which they were generated; having a chronic disease for which the vaccine is not recommended; a lack of evidence about COVID-19 vaccines; and beliefs that these vaccines may contain nanorobots that will track people and control their thinking (Figure 1).

In the analysis, we did not find any correlation on the intention to be vaccinated or not with sex, age, or profession. Only one association with profession was observed (Table 2).

Knowing the number of physicians and nurses who are willing to be vaccinated is also important. Our results show that almost 81% (*n* = 293) of the participant physicians responded that they would be vaccinated against COVID-19. Meanwhile, almost 65% (*n* = 48) of participating nurses responded that they would like to be vaccinated, meaning that 1 out of 3 nurses do not want to be vaccinated, the main reason being the insecurity of the vaccine.

## 4. Discussion

This is the first study to investigate the impact of the COVID-19 vaccine hesitancy in Spain. Our results show that 77.56% (567 participants) of those surveyed were in favour of being vaccinated, well above what televised surveys have reported, which state that more than half of Spaniards (*n* = 55.2%) would prefer to wait to know the effects of a secondary vaccination against COVID-19 [28]. The reasons for refusing to be vaccinated are expected and similar to those found in other studies. However, noticeably, it shows there are no respondents who prefer not to get vaccinated for fear of needles, as presented in other studies [29].

The results could be used to establish communication strategies, since studies have shown that if respondents are doubtful about vaccination, when faced with very strong messages, their doubts may increase instead of diminish [30]. The authorities must assure the population that the development of the vaccine has followed all recommended guidelines for an adequate process of preparation and testing even if it was carried out in a short time, and that this does not mean that it had rushed the quality of the final product. Furthermore, the obligation to vaccinate may have the opposite effect on people with certain doubts about vaccination, although some studies show that incentives or fines could be an effective strategy [31].

Vaccination hesitancy is not only a problem for the general population but also among healthcare workers [32]. The role of healthcare professionals in dispelling doubts is important because their recommendations highly influence the acceptance of vaccination [33]. Careful attention must be paid to this, as during the H1N1 pandemic, primary care doctors were decisive in influencing the population on protective measures [34]. Although COVID-19 vaccine acceptance seems to be higher among doctors than nurses [35], our results show that 34.6% (*n* = 27) of participating nurses responded that they would not be vaccinated versus 17.5% (*n* = 58) of doctors, stating the main reasons as the insecurity of the vaccine and the fear of the vaccine’s side effects. Most nurses are not vaccination immunology skilled, and they would share the same concerns as the untrained population (non-healthcare workers). This is not a new concern; in previous campaigns before COVID-19 the same problem occurred with influenza vaccination [35].

The large number of participants between 56- and 75-years old means that social networks can be used to communicate not only to young adults and/or adolescents but also to older adults (Table 1). On the other hand, the results from participants over 75 years old were scarce, probably due to the lack of Twitter use. It is worth noting here that this age group is also the one that is most influenced by caregivers, family, and health personnel in making decisions about their health and vaccination.

Social networks can be a tool for physicians that could be used to improve patient-physician interactions, enhance patient motivation, drive awareness, provide accurate information, raise timely issues, facilitate the exchange of ideas, frame and reframe health-related questions, engage a larger community, and ultimately produce improved outcomes across health systems [36]. However, it can also cause distraction, contain fake profiles [37], cause difficulty in distinguishing real news from fake news, facilitate cyberbullying, cause unwanted exposure to pornography, and potentially reveal personal information to sexual predators [38]. The possibility of addiction—even in children—must be taken into consideration [39]. Regarding health professionals, concerns about liability, litigation, privacy, lack of time/compensation are found across the spectrum of health professionals [36].

The news media plays an important role in resolving doubts about vaccination. Catalan-Matamoros and Elias noted in their study the possible political burden on the media and the failure to review journalists’ sources who rely on sensationalist media [40].

The results could provide a basis for establishing communication strategies with the Spanish population, but studies with a larger number of participants are needed, perhaps only focused on the causes that could discourage the population from vaccination, to prepare for possible doubts in the population when the COVID-19 vaccine arrives. 

Our findings should be interpreted in light of several limitations. Firstly, attitudes towards COVID-19 vaccination may change over time (especially once health authorities have launched pro-vaccination programs). It is also worth noting the characteristics of the period in which our study was conducted, which may have influenced the results. When the survey was carried out, there was a state of emergency across Spain, which had been reimposed by the government. There was also a nationwide curfew to counter a resurgence of the coronavirus, and no vaccination deployment date had yet been established. Secondly, as a survey-based study, all data was self-reported by participants, and this may have been a source of response bias. However, since the survey was anonymous and participants completed it online without having to interact with any interviewer, this ensured that responses were more honest and accurate. Lastly, the survey we used was a new, non-validated survey tool for determining vaccination intention and attitude towards COVID-19 vaccination. However, we believe that the survey questions we posed were pragmatic in nature and that the responses accurately reflected the sentiment of all groups.

## 5. Conclusions

To maximize the acceptance of the COVID-19 vaccine (when available), it is imperative to recognize the main anti-vaccination beliefs found in our study and other investigations. When the reasons why people do not want to be vaccinated (especially health personnel) are better understood, communication and education strategies can be established, including the use of social networks, to resolve main doubts to achieve a higher vaccination rate and ultimately, the desired herd effect.

To achieve more effective communication, two-way models should be used, which would allow the message to be better perceived by the target group to whom the communication is directed. In other words, the message to be transmitted should be oriented to the needs of the listener. The use of andragogy would be an important advantage.

## Figures and Tables

**Table 1:** Participants’ characteristics and COVID-19 vaccination acceptance.

Age	N	%	95%CI
18–35 years old	86	75.6	(65.4–83.5)
36–55 years old	335	76.4	(71.6–80.7)
56–75 years old	293	79.9	(74.9–84.1)
>75 years old	11	81.8	(49.2–95.4)
**Sex**			
Men	332	79.2	(74.4–83.3)
Women	405	76.5	(72.2–80.4)
**Profession**			
Medicine	274	82.5	(78.1–86.3)
Nursing	51	65.4	(54.2–75.1)
Other health professions	37	68.5	(55.0–79.5)
Non-health professions	166	76.1	(70.0–81.3)
Unemployed	39	79.6	(66.0–88.7)

Six missing for age and four for sex are not included in the description.

**Table 2:** Association between predisposition to be vaccinated and occupation.

Occupation	PR	95%CI	*p*-Value
Physician	1		
Nurse	1.146	(1.052–1.249)	0.002 **
Other health worker	1.119	(1.012–1.238)	0.028 *
Other non-health worker	1.054	(0.995–1.117)	0.071
Unemployed	1.025	(0.927–1.133)	0.628

Note: *: *p* < 0.05, **: *p* <0.01. CI: confidence interval. PR: Poisson Regression.

**Figure A1:**
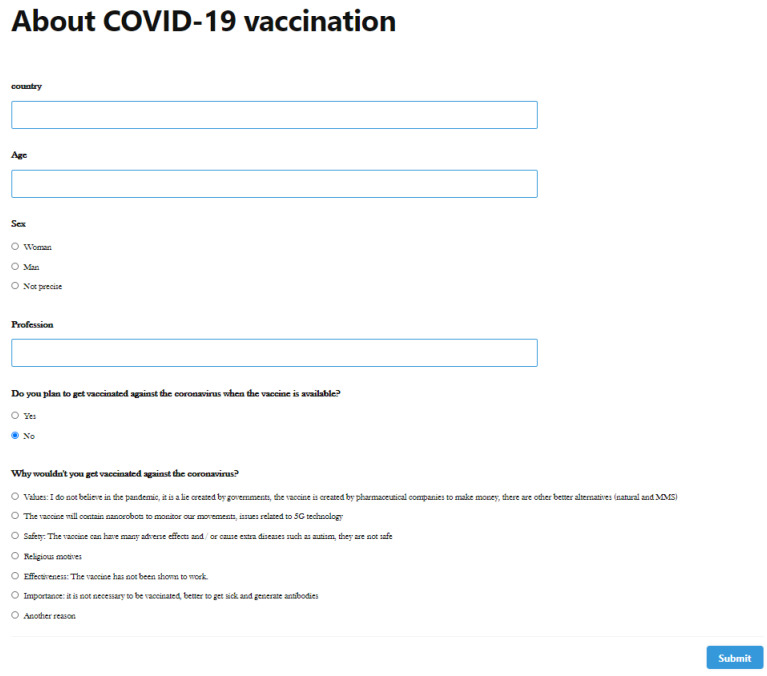
Questionnaire sent in Twitter.
